# Simultaneous Determination of Dissolved Organic Carbon and Total Dissolved Nitrogen on a Coupled High-Temperature Combustion Total Organic Carbon-Nitrogen Chemiluminescence  Detection (HTC TOC-NCD) System

**DOI:** 10.1155/JAMMC.2005.240

**Published:** 2005

**Authors:** Xi Pan, Richard Sanders, Alan D. Tappin, Paul J. Worsfold, Eric P. Achterberg

**Affiliations:** 1 National Oceanography Centre Southampton University of Southampton European Way Southampton SO14 3ZH UK; 2 School of Earth Ocean and Environmental Science University of Plymouth Plymouth PL4 8AA UK

## Abstract

The marine biogeochemistries of carbon and nitrogen have come under increased scrutiny because of their close involvement in climate change and coastal eutrophication. Recent studies have shown that the high-temperature combustion (HTC) technique is suitable for routine analyses of dissolved organic matter due to its good oxidation efficiency, high sensitivity, and precision. In our laboratory, a coupled HTC TOC-NCD system with a sample changer was used for the automated and simultaneous determination of dissolved organic carbon (DOC) and total dissolved nitrogen (TDN) in seawater samples. TOC control software was used for TOC instrument control, DOC data acquisition, and data analysis. TDN data acquisition and manipulation was undertaken under LabVIEW. The combined system allowed simultaneous determination of DOC and TDN in the same sample using a single injection and provided low detection limits and excellent linear ranges for both DOC and TDN. The risk of contamination has been remarkably reduced due to the minimal sample manipulation and automated analyses. The optimised system provided a reliable tool for the routine determination of DOC and TDN in marine waters.


Journal of Automated Methods & Management in Chemistry, **2005** (2005), no. 4, 240–246
© 2005 Hindawi Publishing Corporation

### Simultaneous Determination of Dissolved Organic Carbon and Total Dissolved Nitrogen on a Coupled High-Temperature Combustion Total Organic Carbon-Nitrogen Chemiluminescence Detection (HTC TOC-NCD) System

Xi Pan,[1] Richard Sanders,[1] Alan D. Tappin,[2] Paul J. Worsfold,[2] and Eric P. Achterberg[1]

[1]*National Oceanography Centre Southampton, University of Southampton, European Way, Southampton SO14 3ZH, UK*
[2]*School of Earth, Ocean and Environmental Science, University of Plymouth, Plymouth PL4 8AA, UK*

*Received 20 April 2005; Accepted 12 May 2005*

The marine biogeochemistries of carbon and nitrogen have come under increased scrutiny because of their close involvement in climate change and coastal eutrophication. Recent studies have shown that the high-temperature combustion (HTC) technique is suitable for routine analyses of dissolved organic matter due to its good oxidation efficiency, high sensitivity, and precision. In our laboratory, a coupled HTC TOC-NCD system with a sample changer was used for the automated and simultaneous determination of dissolved organic carbon (DOC) and total dissolved nitrogen (TDN) in seawater samples. TOC control software was used for TOC instrument control, DOC data acquisition, and data analysis. TDN data acquisition and manipulation was undertaken under LabVIEW. The combined system allowed simultaneous determination of DOC and TDN in the same sample using a single injection and provided low detection limits and excellent linear ranges for both DOC and TDN. The risk of contamination has been remarkably reduced due to the minimal sample manipulation and automated analyses. The optimised system provided a reliable tool for the routine determination of DOC and TDN in marine waters.

### 1. INTRODUCTION

Global change and global warming have become dominant issues in environmentalscience. The increased use of fossil fuels in the postindustrial period has resulted in enhanced emissions of greenhouse gases, including $CO_2$. The oceans form an important sink of atmospheric $CO_2$ through both the physical and biological pumps [1]. As part of the biological pump, atmospheric $CO_2$ is fixed by primary producers in the surface ocean and planktonic detritus is transferred to the deep ocean following cell death. This process transfers atmospheric carbon to the deep ocean for medium (1000′ s of years) to long (millions of years) time periods. Both in the surface and deep ocean, particulate planktonic detritus is mineralised by marine heterotrophic organisms and dissolved organic carbon and nutrients are released. Furthermore, DOC is released into the surface waters via phytoplankton exudation and cell lysis [2]. As a consequence of these processes the concentrations of DOC are closely related to primary production and are highest in the surface waters and decrease markedly through the thermocline to low levels in deep oceanic water. The importance of oceanic DOC is indicated by Siegenthaler and Sarmiento [3], who reported that the amounts of carbon in oceanic DOC ($\sim 700 \times 10^{15}$ g C) are similar to those of atmospheric $CO_2$ ($\sim 750 \times 10^{15}$ g C). Thus, a net oxidation of merely 1% of the oceanic DOC will be sufficient to generate $CO_2$ fluxes, which are greater than those produced annually by fossil fuel combustion [4]. An improved understanding of the distribution and cycling of marine DOC is therefore important for a global assessment of fluxes and reservoirs of carbon.

Total dissolved nitrogen (TDN) comprises dissolved organic nitrogen (DON) and dissolved inorganic nitrogen (DIN) in the forms of nitrate, nitrite, and ammonium. The DON fractions have historically been ignored because they were considered to be biologically inert [5]. Recent studies, however, indicated that DON concentrations account for as much as 40%–70% of the TDN pool in surface seawater and an important fraction of DON is readily available to the microbial community [2]. Nitrogen compounds are introduced into the marine environment mainly by riverine and atmospheric inputs and nitrogen fixation. In coastal water,

Correspondence and reprint requests to Eric P. Achterberg, National Oceanography Centre Southampton, University of Southampton, European Way, Southampton SO14 3ZH, UK; E-mail: eric@noc.soton.ac.uk.

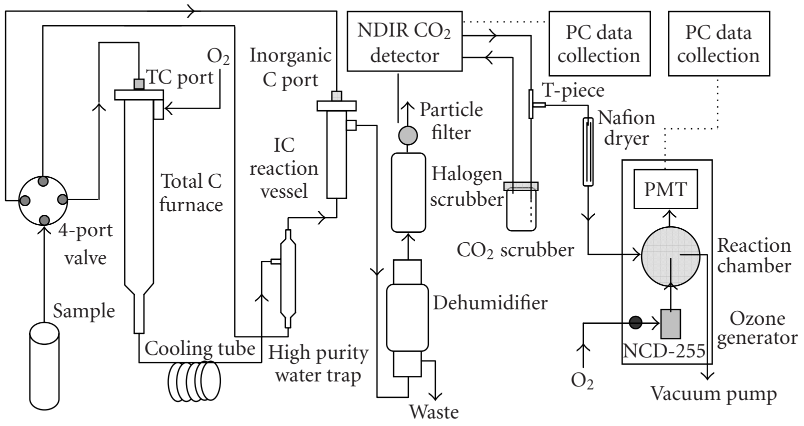

Figure 1: A schematic of the Shimadzu TOC 5000A total carbon analyser coupled with the Sievers NCD 255 nitrogen chemiluminescence detector (adapted from Badr et al, 2003).

excess nitrogen inputs lead to eutrophication and harmful algal blooms. However, in the open ocean the surface waters are typically subject to nitrogen limitation. The majority of studies of nitrogen in the marine environment have only determined DIN (using standard colorimetric techniques), however the omission of the DON fractions may lead to an important underestimation of nitrogen available to the microbial community [2].

Different analytical techniques have been applied for the determination of DOC in marine waters, including UV oxidation [6], wet chemical oxidation using persulphate [7], and high-temperature combustion (HTC [8]). The $CO_2$ produced in these techniques during the oxidation of organic carbon is quantified using nondispersive infrared detection. The HTC technique is currently the most widely used approach [9] because of its good oxidation efficiency, high sensitivity, and precision.

As no analytical technique exists for direct DON measurements, this variable is determined through separate DIN (using colorimetric techniques) and TDN analyses. A commonly used technique for TDN analyses involves wet chemical oxidation using persulphate digestion and UV oxidation followed by colorimetric nitrate analysis [10]. More recently, high-temperature combustion of TDN followed by chemiluminescence detection of NO has become the dominant technique [10, 11]. In this paper we describe the development of hardware and software, and the application of a coupled analytical system for automated and simultaneous determination of DOC and TDN in marine waters. This coupled online approach allows a high sample throughput and facilitates carbon and nitrogen clean operating procedures, and the detection systems exhibit a high sensitivity, precision, and minimal interference problems.

### 2. EXPERIMENTAL

### 2.1. Reagents and samples treatment

Plasticware and glassware were cleaned by soaking in decon solution (2% v/v, Analar grade, VWR Ltd, England) for 24 hours followed by HCl (10% v/v, Analar grade, VWR Ltd, England) for 24 hours. De-ionised water (Milli-Q, Millipore Inc, England) was used for thorough rinsing between each cleaning step. Glassware was then combusted at 450°C for 6 hours to remove remaining organic residues. Samples were collected using glass bottles. In order to minimise the influences of biological degradation, samples were filtered immediately upon collection using ashed glass fibre filters (0.7 $\mu$M pore size, 47 mm diameter, Whatman GF/F, England) with an ashed glass filtration unit (Nalgene, US). Flame-sealable glass ampoules (Adelphi Ltd, England) were employed for sample storage. Prior to closing the ampoules, HCl (50% v/v, Analar grade, VWR Ltd, England) was added to acidify the samples to pH 2. A potassium hydrogen phthalate and glycine (both Analar grade, VWR Ltd, England) mixture (C : N atom ratio = 6 : 1) was made up for calibration of the coupled TOC-NCD instrument.

### 2.2. Coupled TOC-NCD system

In our laboratory, HTC measurements of DOC and TDN were performed using a Shimadzu TOC 5000A total carbon analyser (Shimadzu Corp, Japan) coupled with a Sievers NCD 255 nitrogen chemiluminescence detector (Sievers Instruments, Inc, US). Ancillary instrumentation included an autosampler (ASI 5000A, Shimadzu Corp, Japan) and a two-stage vacuum pump (no 8, Edwards High Vacuum Ltd, England). Carbon-free, high purity (99.999%) oxygen gas (BOC gas, England) was applied as carrier gas. Figure 1 shows a schematic diagram of the coupled HTC TOC-NCD system. A full analytical measurement cycle consists of two steps.

*Step* 1. Acidified sample was sparged using pure oxygen for a period of 8 minutes (75 mL min$^{-1}$) to remove inorganic carbon compounds and then injected into the furnace of the TOC instrument. The carrier gas pushed the sample into the combustion column, which was heated to 680°C and filled with a catalyst (aluminium oxide coated by 0.5% of platinum (Shimadzu Corp, Japan)). Organic carbon and nitrogen compounds, and ammonia in the sample were oxidisedto

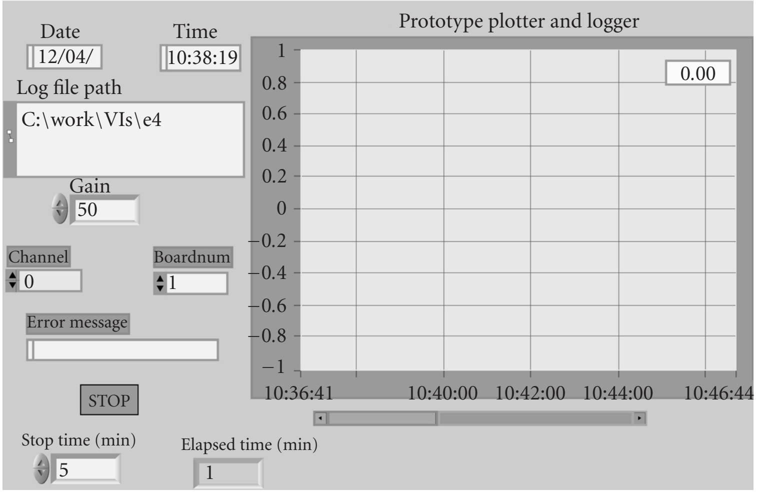

Figure 2: LabVIEW graphical user front panel for the automated virtual NCD instrument.

$CO_2$, NO, and $H_2O$, whereas nitrate and nitrite were reduced to NO.The gas stream was pushed in turn through a cooling tube, an electronic dehumidifier to remove water vapour, and a Cu-based halogen scrubber for gas purification. Then, the dried, purified gas stream was drawn into the sample cell housed in a nondispersive infrared (NDIR) gas analyser, where $CO_2$ in the gas stream was detected by the NDIR detector. The produced analogue signals were amplified by the amplifier and converted to pulse frequency by the V/F converter.

*Step* 2. The gas stream was drawn out of the TOC instrument with the aid of the vacuum pump, passed through a Nafion membrane dryer (Perma Pure Inc, US) in order to remove any remaining water vapour, and then routed to the NCD reaction chamber. The additional drying step using the Nafion dryer is important as moisture quenches the chemiluminescence reaction and leads to tailing peak [5]. The vacuum pump produced operating pressures of 2–10 torr in the reaction cell. The TDN determination relied on the chemiluminescence reaction of NO and $O_3$. NO generated from Step 1 reacted with $O_3$ produced by the ozone generator housed in the NCD instrument to give rise to the radical species $NO_2{}^*$, which emitted quantifiable energy (light) upon decay to its ground state:

$$NO + O_3 \longrightarrow O_2 + NO_2{}^* \longrightarrow NO_2 + h\nu. \qquad (1)$$

The reaction of NO and $O_3$ occurred in front of the photo-multiplier tube (PMT). The emitted light ($h\nu$) was collected by the PMT and converted to a voltage signal stoichiometrically proportional to the total nitrogen concentrations in the sample.

Samples for nitrate and nitrate (DIN) for a North Sea research cruise fieldwork were analysed using standard colourimetric techniques [12]. Salinity was determined using an in situ conductivity probe as part of a standard ship-board CTD system.

### 2.3. DOC data acquisition and manipulation

TOC instrument control was undertaken using the TOC control (5000/5000A & 5050/5050A) software (version 1.05.01, Shimadzu Corp, Japan) installed in a desktop computer linked to an RS-232 board (PC-15N) (Shimadzu Corp, Japan) placed in the rear of the Shimadzu TOC instrument via an AWM E101344 style 2464 28AWG cable. This software was also capable of data acquisition and manipulation. Pulse frequency was recorded and quantified as peak area by the Shimadzu Model CR6A Chromatopac computing integrator. Following the analyses of calibration standards, the software allowed for automated calculations of DOC concentrations in the samples. The results of the DOC concentration calculations, including statistic analysis are displayed in a real-time window and stored on the computer's hard-disk.

### 2.4. TDN data acquisition

The analogue signals from the Sievers NCD instrument were collected by a CIO-DAS08/JR/16 A/D card (Talisman Electronic Ltd, England) installed in a desktop computer through a female 37-pin, D-type connector (Talisman Electronic Ltd, England). An interface box was built to link the analogue output of the NCD to the A/D card. A TCIO-MINI37 screw terminal board (Talisman Electronic Ltd, England) was placed in the interface box. A one metre long TC37FF-5 cable (Talisman Electronics Ltd, England) linked the terminal board and the D-type connector on the A/D card.

A LabVIEW graphical programming environment was used to design a virtual instrument (VI) with a flexible user interface for the TDN data acquisition and manipulation. The VI software was developed in collaboration with Ruthern Instruments (Bodmin, UK) and written in LabVIEW version 6 (National Instruments Corp, US). The digital signals from the A/D card were acquired by the VI. The LabVIEW front panel contains ready-to-use switches, buttons, and controls to start the data acquisition, define the signal gain, and stipulate the stop time of the data collection (Figure 2).

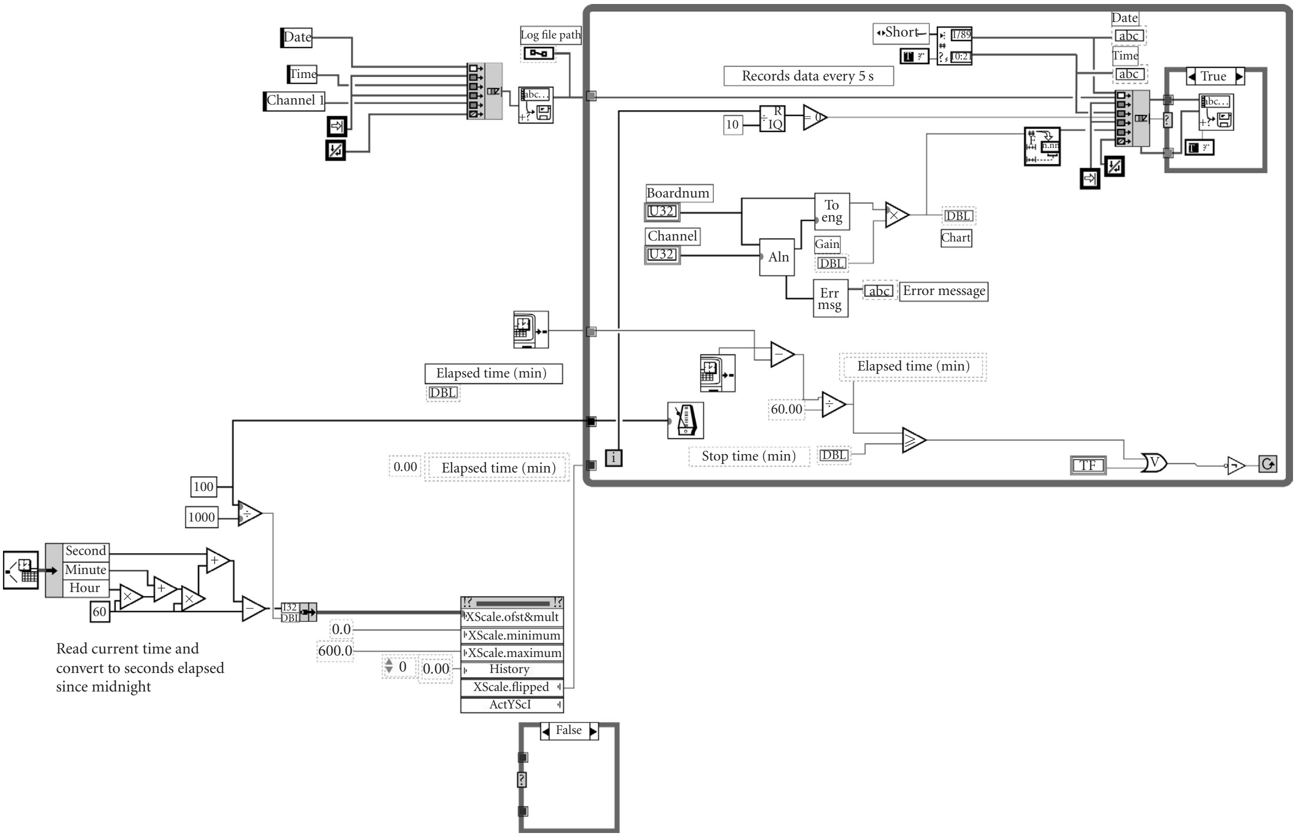

Figure 3: Graphical code for NCD instrument control.

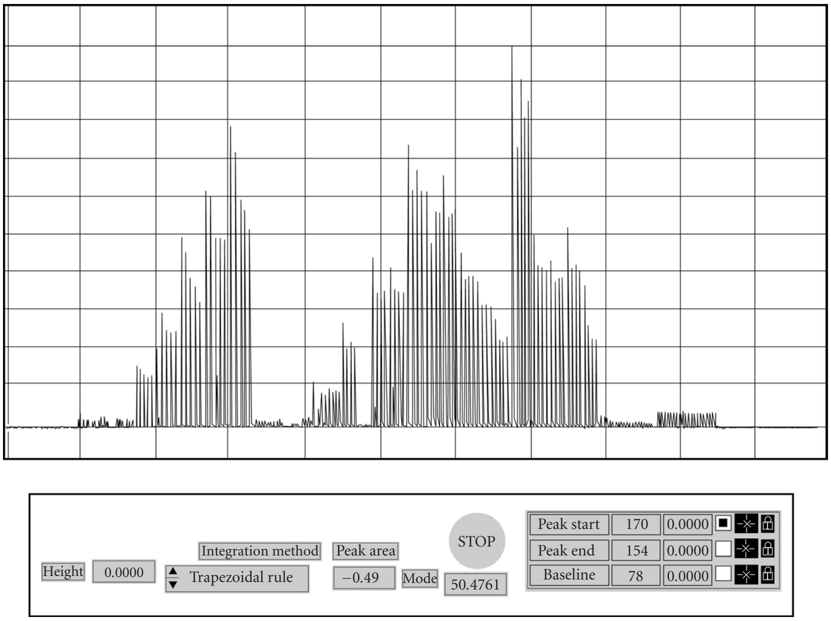

Figure 4: LabVIEW graphical user front panel for data analyses.

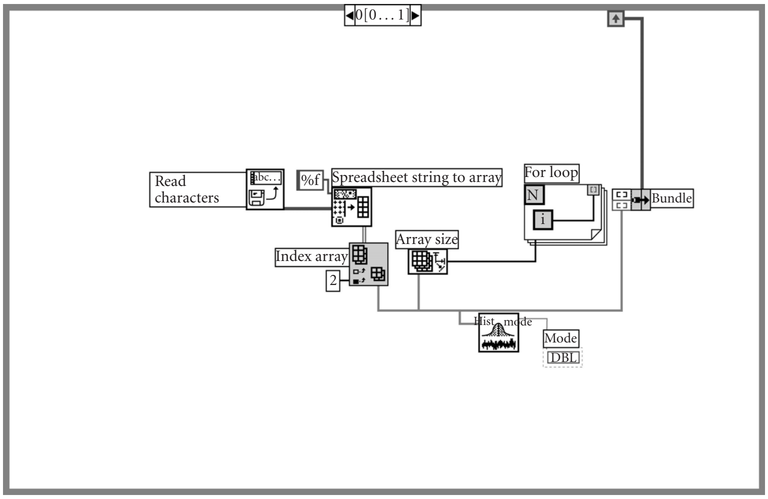

(a)

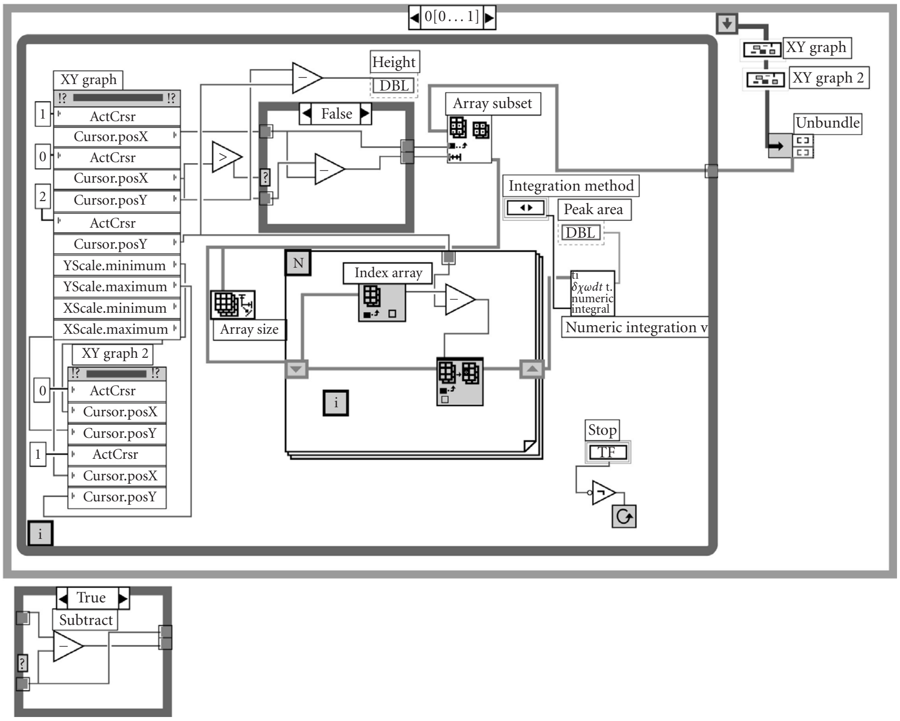

(b)

FIGURE 5: Graphical code for data analysis.

The nitrogen signals are displayed in real-time in the box on the front panel. The LabVIEW VI wiring diagram for the TDN data acquisition is presented in Figure 3, showing the data entry, analysis definitions, and stop procedure.

Once the coupled TOC-NCD instrument is running, data acquisition can be started instantaneously by depressing the run icon from the LabVIEW environment element bar (Figure 2). The processes can be halted by depressing the abort icon, alternatively the data acquisition is stopped automatically afterthe time period indicated in the "stop time" window on the front panel (Figure 2). A real-time window displayed on the right-hand side of the front panel visualises the data acquisition process. Data is stored on the hard disk of the computer.

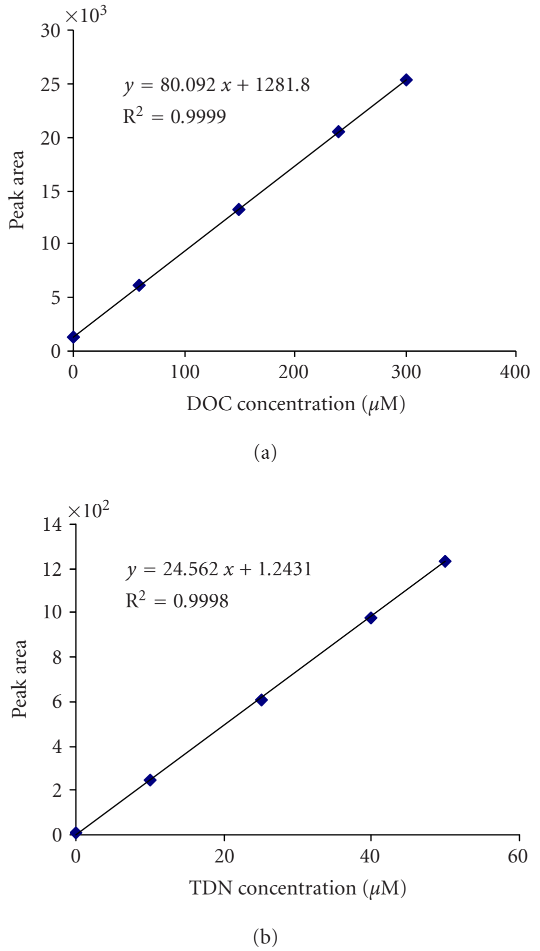

Figure 6: Calibration graphs of DOC and TDN.

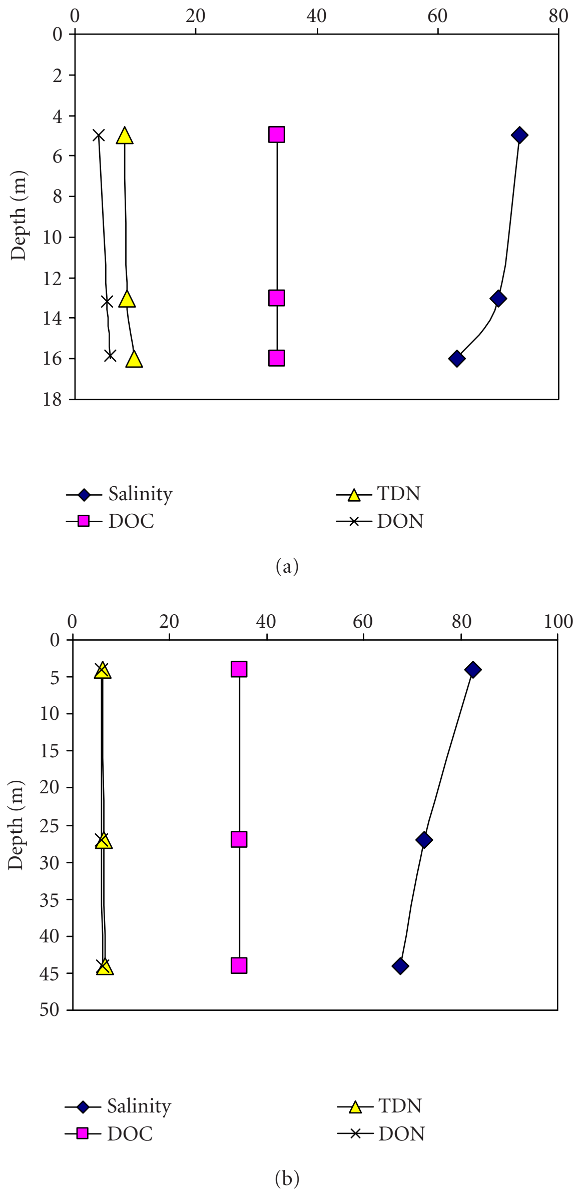

Figure 7: (a) Depth distribution of salinity, and DOC, TDN, and DON (in $\mu$M) at near shore station (station 7) in North Sea (53° 32.7′N, 0° 23.5′E), sampled in September 18, 1999. (b) Depth distribution of salinity, and DOC, TDN, and DON (in $\mu$M) at offshore station (station 26) in North Sea (54°12.7′N, 1°36.7′E), sampled in September 21, 1999.

### *2.5. TDN data manipulation*

An additional LabVIEW VI was designed to allow quantification of TDN peak area. Figure 4 shows the graphical user front panel of the virtual data manipulation instrument. The virtual data manipulation instrument is flexible, and allows positioning of the baseline and a user-controlled definition of the peak start and peak end position. The automated calculation of peak area can be undertaken using a range of different integration methods. The approach typically used for our TDN peak area determinations applies the trapezoidal rule, which is a mathematic method for approximation of a definite integral by evaluating the integrand at finitely many points. The LabVIEW VI wiring diagram for the TDN data manipulation is presented in Figure 5, showing the data entry and integration approach.

### 3. ENVIRONMENTAL APPLICATION

Examples of standard solution graphs for DOC and TDN determinations are presented in Figure 6, both of which showed excellent linearity. Linear ranges were estimated to be 8–600 $\mu$M C and 0.3–100 $\mu$M N, respectively. The limit of detection ($3\sigma_{\text{blank}}$) was 8.0 $\mu$M Cand 1.0 $\mu$M N. For the combined HTC TOC-NCD in our laboratory, the coefficient of variation was less than 1.5% (3–5 repeat injections) and de-ionised water blank was lessthan 10 $\mu$M TOC and about 1.0 $\mu$M TDN. For the quantitative validation and accreditation of analytical measurements, a deep ocean water supplied by the Hansell Laboratory (University of Miami, US) was used as a certified reference material (CRM). The CRM was analysed with every sample run and the results (44–46 $\mu$M DOC and 21.5 $\mu$M TDN) obtained using the combined HTC TOC-NCD system showed an excellent agreement with the certified values (44.3 $\mu$M DOC and 21.2 $\mu$M TDN).

The combined TOC-NCD system has been used for analysis of samples collected during the IMPACT cruise, conducted on the North Sea in September 1999. Figures 7a and 7b show depth profiles of salinity, DOC, TDN, and DON for inshore (station 7) and offshore waters (station 26) of the North Sea.The DOC concentrations ranged approximately between 65–88 $\mu$M at these stations. The highest DOC concentrations were observed in the surface waters, most likely as a result of enhanced primary productivity in these waters. The TDN concentrations were low (below 10 $\mu$M), indicating nitrogen uptake by primary producers and dilution of coastal waters with cleaner Atlantic waters flowing into the western North Sea from the north. Dissolved organic nitrogen constituted between 48% and 60% of the TDN at station 7, and between 91% and 94% at station 26. This shows that DON forms the major part of the dissolved nitrogen pool in the North Sea. The inshore station (station 7) is more influenced by DIN inputs from the Humber Estuary, and hence has a lower percentage of DON as part of TDN compared with the offshore station (station 26). The DOC, TDN, and DON concentrations observed in the present study agree well with previous work in other marine waters [2]. However, the DON/TDN ratio at station 26 was higher than the reported average range [2], indicating the high uptake of inorganic nitrogen species by microbial organisms in the highly productive North Sea, with subsequent production of DON.

### 4. CONCLUSIONS

The combined HTC TOC-NCD system provided excellent linearity and precision. The results of deep oceanic water CRMs for the system showed a good agreement with the certified values. TOC control software was capable of automated peak area recognition and quantification, concentration calculation, and statistical analysis. The developed LabVIEW VIs allowed flexible TDN data acquisition and manipulation. This optimised system allowed simultaneous analysis of DOC and TDN in seawater samples using a single injection of the same sample and hence involved a minimum of sample handling and risk of contamination. The limit of detection makes it feasible to determine DOC and TDN in all marine environments. The optimised methodology will allow the studies of the distribution and behaviours of DOC and TDN/DON in marine ecosystems and improve our understanding of marine biogeochemistry of carbon and nitrogen.

### ACKNOWLEDGMENTS

Xi Pan acknowledges the financial support by the University of Southampton. Mr John Woods of Ruthern Instruments is gratefully acknowledged for his hardware and software support. The captain and crew of the *RRS Challenger* are thanked for outstanding support on the North Sea IMPACT cruise.

### REFERENCES

[1] J. Houghton, *Global Warming: The Complete Briefing*, Cambridge University Press, Cambridge, UK, 3rd edition, 2004.

[2] D. A. Bronk, "Dynamics of DON," in *Biogeochemistry of Marine Dissolved Organic Matter*, D. A. Hansell and C. A. Carlson, Eds., pp. 153–247, Academic Press, San Diego, Calif, USA, 2002.

[3] U. Siegenthaler and J. L. Sarmiento, "Atmospheric carbon dioxide and the ocean," *Nature*, vol. 365, no. 6442, pp. 119–125, 1993.

[4] J. I. Hedges, "Global biogeochemical cycles:progress and probles," *Marine Chemistry*, vol. 39, pp. 67–93, 1992.

[5] T. W. Walsh, "Total dissolved nitrogen in seawater: a new-high-temperature combustion method and a comparison with photo-oxidation," *Marine Chemistry*, vol. 26, no. 4, pp. 295–311, 1989.

[6] F. A. J. Armstrong, P. M. Williams, and J. D. H. Strickland, "Photooxidation of organic matter in seawater by ultraviolet radiation, analytical and other applications," *Nature*, vol. 211, pp. 481–483, 1966.

[7] N. Ogura, "The relation between dissolved organic carbon and apparent oxygen utilization in the Western North Pacific," *Deep-Sea Research and Oceanographic Abstracts*, vol. 17, no. 2, pp. 221–231, 1970.

[8] Y. Suzuki, Y. Sugimura, and T. Itoh, "A catalytic oxidation method for the determination of total nitrogen dissolved in seawater," *Marine Chemistry*, vol. 16, no. 1, pp. 83–97, 1985.

[9] G. Spyres, M. Nimmo, P. J. Worsfold, E. P. Achterberg, and A. E. J. Miller, "Determination of dissolved organic carbon in seawater using high temperature catalytic oxidation techniques," *TrAC Trends in Analytical Chemistry*, vol. 19, no. 8, pp. 498–506, 2000.

[10] J. H. Sharp, A. Y. Beauregardt, D. Burdidge, et al., "A direct instrument comparison for measurement of total dissolved nitrogen in seawater," *Marine Chemistry*, vol. 84, no. 3-4, pp. 181–193, 2004.

[11] E.-S. A. Badr, E. P. Achterberg, A. D. Tappin, S. J. Hill, and C. B. Braungardt, "Determination of dissolved organic nitrogen in natural waters using high-temperature catalytic oxidation," *TrAC Trends in Analytical Chemistry*, vol. 22, no. 11, pp. 819–827, 2003.

[12] T. R. Parson, *A Manual of Biological and Chemical Methods for Seawater Analysis*, Pergamon Press, Oxford, UK, 1990.

